# Textile Supplies for Hospitals

**Published:** 1916-01-08

**Authors:** 


### TEXTILE SUPPLIES FOR HOSPITALS

### II. The Basis of Values.

Excuses can be found for the prevalence of question-begging ideas concerning textile supplies and for writers upon the subject who have perpetuated notions such as that "good materials have always a firm, strong selvage." There is the manifest excuse that in any given case—and with the means at disposal—the principles of knowledge may be difficult to apply. None the less is it the fact that the value of textiles is not to be gauged by any index feature of that kind, but only by the materials and workmanship that have entered into them. If a fabric is good for the purpose to which it is put, that is because in the first place the raw material is suitable. The raw material is the true determinant of quality, and its suitability can be expressed in definite terms. The fibres are of a certain average length and fineness, of a certain strength individually, and a certain combining power. They have a measure of absorbency and of conductivity; they have a natural absence or presence of coloration, with perhaps an inherent lustre. They are, besides, of a certain chemical constitution, and although it is not the habit of those who buy textile fibres for manufacturing consumption to put the material to the microscopical and other tests that reveal its precise nature, the minute differences between one sample and another are carefully observed, and surprisingly minute variations discover themselves to the practised eye and the cultivated touch. The respective capacities of the different raw materials call for treatment in detail, but the immediate business is with general principles.

### The Comparison of Cloths.

Taking any two samples of fabrics in hand and accepting as a fact the general suitability of the raw material within them, there are further points about them to be observed. They consist of bundles of fibre twisted into yarn of one diameter or another, and in the process of weaving these threads are intercrossed. It is the case that raw material of one quality will spin higher than material of another, or, in other words, produce a uniform thread of greater fineness. The fibre that spins highest is worth the more in the market, and as fine-spinning is a lengthier process than coarse-spinning, the finer-spun yarn—other things equal—is the more valuable of the two. The ratio of fineness is expressed not in sizes, but in length per lb., and as yarns of one class and another may run anything between a few hundred yards and a hundred miles to the pound, the extremes of difference are immediately appreciable, although small differences are not perceptible to the untrained eye. Coarse yarns occupy more room within a woven fabric than finer ones, and as they yield fewer yards to the pound they bring up the weight of the goods into which they are made.

### Weight as an Index of Value.

Weight is one of the most easily ascertainable facts about woven goods, and it may be expressed as a weight per pair, as in blankets, or a weight per piece of a specified number of yards, or a weight per yard of known width. Weight is one index of value, and a factor necessary to be taken into account in comparing one fabric with another. The heavier of two samples is not necessarily the more valuable or the more suitable for the purpose, because weight may be gained in either of two ways. The simplest way to make a fabric heavy is to weave it from thick yarn with few threads to the square inch. A fabric of equal weight, containing more threads within a given area, is naturally the more expensive of the two, and its finer yarn probably involves the use of a higher grade of raw material, and the costs, both of spinning and of weaving, are inevitably more. Thus, in making a close comparison between woven samples, regard is paid to the weight and to the threads, which can be counted in a measured space of one-quarter, one-half, or one inch. A small instrument, the "piece-glass" or "linen-prover," is sold for the purpose, consisting of a magnifying glass mounted in a folding metal frame, and having a measured slot at the foot, through which the individual threads are counted. Threads cannot be counted readily in all fabrics, even with the aid of the glass, but the principle remains. It can be taken as a universal rule that the clue to the relative commercial values of woven goods lies in the kind and quality of the raw materials employed; in the weight of the goods and the number of threads to the inch.

### The Influence of Structure.

Felts are fabrics which may be made without spinning or weaving simply by taking advantage of the capacity inherent in some fibres of interlocking under favourable conditions. Knitted goods are the outcome of the successive looping of one or more threads, but woven cloth is only to be produced by the intercrossing of two sets of threads worked at right angles. The longitudinal threads are the warp, and the transverse threads of the weft intersect them either alternately or in a rotation, producing some distinct pattern. Warp and weft are not necessarily of the same quality as each other or even of the same fibre. As it is subjected to greater strain in weaving and, usually, in wear the warp is normally made the stronger of the two, and this strength is obtained either by employing fibres which are individually rather longer than for weft or by giving the warp yarn a higher degree of twist. It is easy to show by screwing fibrous material between the fingers that added twist gives added strength up to a point. In practice the advantage of hard twisting is limited by the

Note.—The first article in this series appeared on page 255 of The Hospital for December 18, 1915. The next article will be on Textile Raw Materials and their Properties.

consequent slowing-down of machines and by the hardness imparted to the material. It is twist that lends fibres cohesion and enables them to be woven, but excess of twist is not universally desirable for its own sake.

On detaching threads of yarn from a fabric and rolling them between the fingers in the direction contrary to the twist, the yarn shreds away to individual fibres if the yarn is of single strand. Yarns are the stronger for being two-fold, and in that case upon unrolling the strands appear separately. Two-fold yarn is used in weaving where strength rather than fulness and softness is wanted, and when found is present generally in the warp. Strength is a factor in most of the cloths required in a hospital, but is not a sole desideratum in most cases, and strength, measured either by direct breaking strain or resistance against attrition, may be bought at the expense of properties no less desirable in themselves. Warmth-giving properties are manifestly best consulted by a free and open arrangement of the fibres that is the very opposite of the tightly-screwed condition most favourable to strength. Absorbency of fluids owes something to a capillary attraction which makes spun yarn preferable to a disjointed mass of fibres, but absorption is not indefinitely promoted by hard twisting. The two respects are chief ones, and they are best satisfied by the employment of goods made from fibres of a sufficient average length to make hard twisting unnecessary to promote wear.

### The Principle of Warmth.

The suitability of fabrics for their purpose is as much a matter of how they are made as of the raw materials composing them. Such as are intended for warmth challenge comparison with the provision that is made by Nature. Animals and birds are given a covering of non-conducting fibres, arranged at a slant so that they effectually imprison a layer of warm air next to the body. We go to one of these animals, the sheep, for the best non-conducting fibre for warm clothes, but clothes of the same wool and the same weight are not equally warm in wear. The explanation lies in the arrangement of the fibres made in the course of manufacture, and it is even found that under a favourable arrangement fibres not remarkable for their non-conducting properties can be brought into service for warmth. The leading example is the cotton blanket, or its equivalent in piece-goods, the notorious flannelette. Teased in such a manner as to contain air within its interstices, even cotton can be made to do duty for wool. The instance serves as an extreme to illustrate a point which has other exemplifications. It is a matter of common experience that in pure warmth-giving the finest wool is superior to the middling-fine, and that in turn medium is superior to coarse. The fact can hardly have any other explanation than that by reason of the fineness of their fibres fabrics of the finest wool enchain warmed air more effectually. There is little but rough experience to go upon, and the whole question of the relations of heat-conserving properties to value in money deserves more attention than it has had, but meantime the prime object should not be ignored. Warmth being the objective, as much of it as possible should evidently be got for the money.

### The Principle of Absorbency.

In regard to surgical dressings, of which the absorbency is unmistakably a prime feature, some amount of scientific work has been done, and it has been demonstrated to satisfaction that short-fibred dressings hold more fluid than the long-fibred. The point has its bearing upon such hospital purchases as towels, which are fabrics bought for their absorbent properties primarily, and secondarily for their wear. The difference between towels of approximately equal fibres are small enough to be dismissed, but the hospitals are buyers of linen towels made from flax of very long fibre and cotton towels made from one of the shortest fibres in textile use. In such a case the most pointed testimony would be that of the washtub rather than of laboratory research, and it is unlikely that its records have gone neglected. The fact, however, seems to deserve mention that both cottons and linens are in use, and that in sheer capacity for absorption the materials are unequal. The literal composition of a towel is one thing and its actual working out another. To take a crude case—the distinction between a plain towelling and one with a dense, bushy pile has to be recognised.

### Certain Tests.

In no instance can the joint effects of the material and the method of its employment be ignored, so that there is peculiarly little room for facile generalisations. The good selvages that have been associated in some experience with hard-wearing goods are mere excrescences upon the body of the fabric, and may be varied to taste, as is, indeed, done to satisfy the prejudices of the China market. There are not many popular conceptions about fabrics that are not more often untrue than true. The ideas that cloth is good because it is hard or stiff, or because it can be so manipulated as to emit a crack under the finger and thumb, are all of strictly limited validity, and the less dependence that is placed upon them the better. For almost any purpose, however, it is desirable that a fabric should be reasonably strong, and there is no difficulty in making a simple test. Cloth is strong enough for most ordinary uses when no break or hole can be made in it by exerting pressure with the thumbs. The fabric is taken under the two thumbs, the fingers being clenched and the knuckles in face of each other, with the thumbs close together. Steady downward pressure with the thumbs is then used, and in this way a fair notion of the comparative tensile strengths of light fabrics can be obtained. The method discloses any tendency of the threads to slip over each other, and it is to be distinguished carefully from a violent tearing in which an uncertain amount of force is used.

